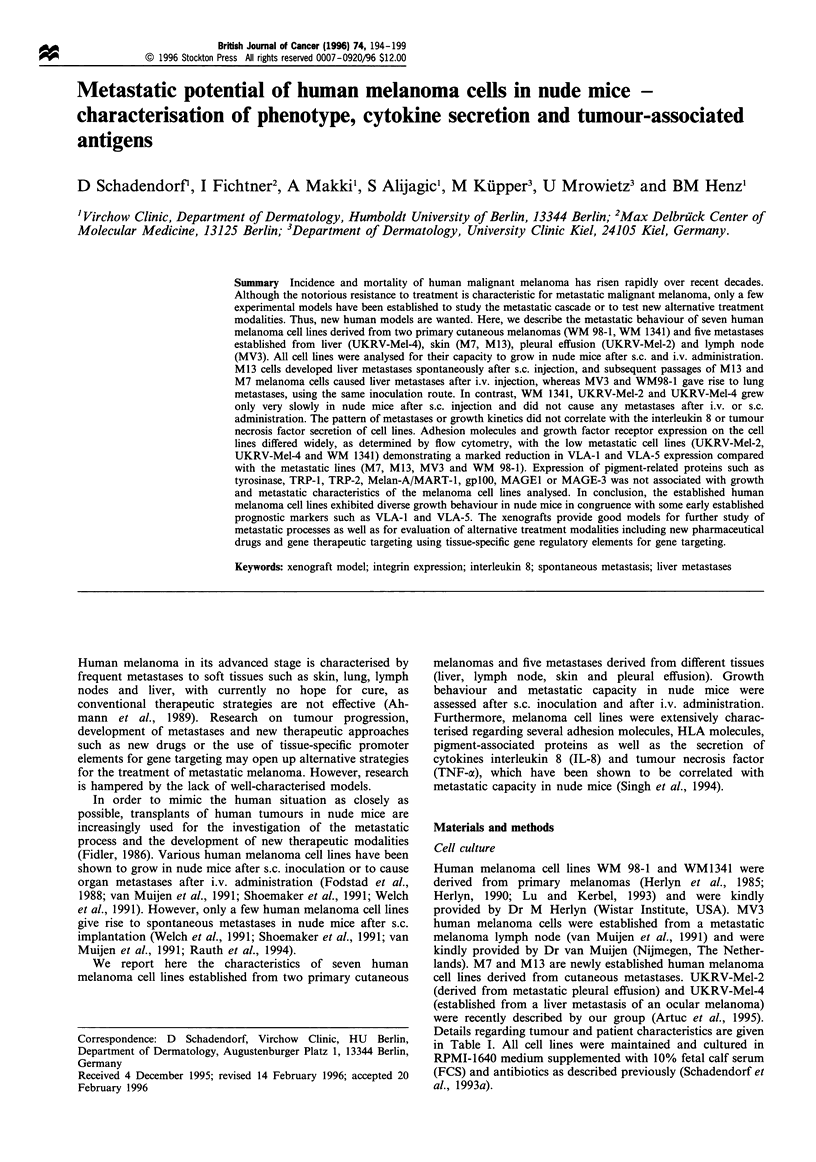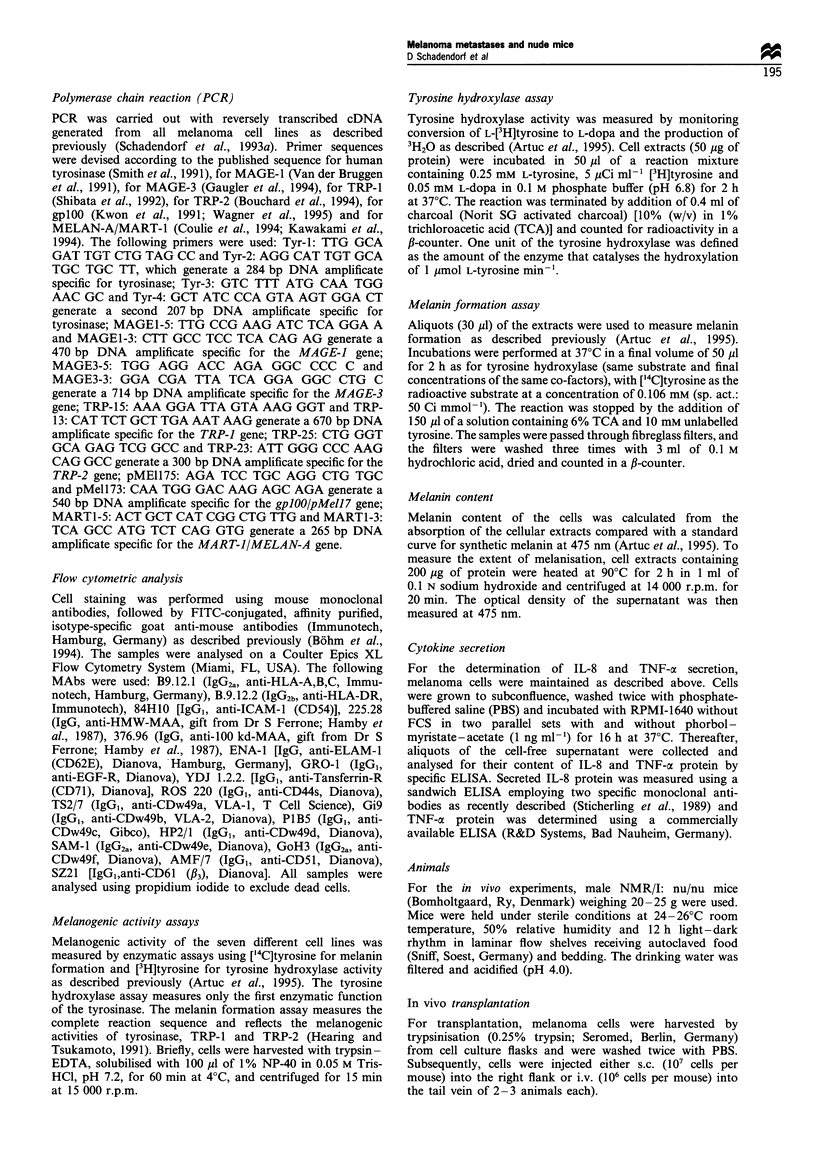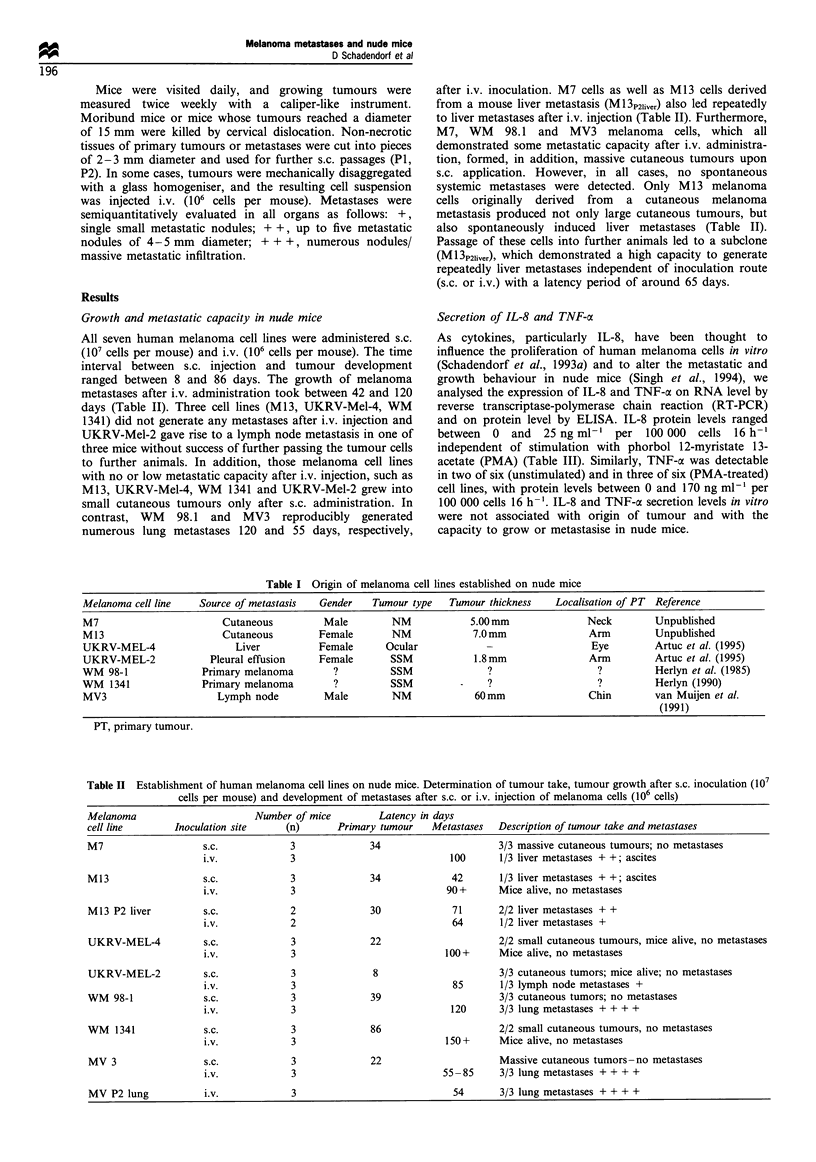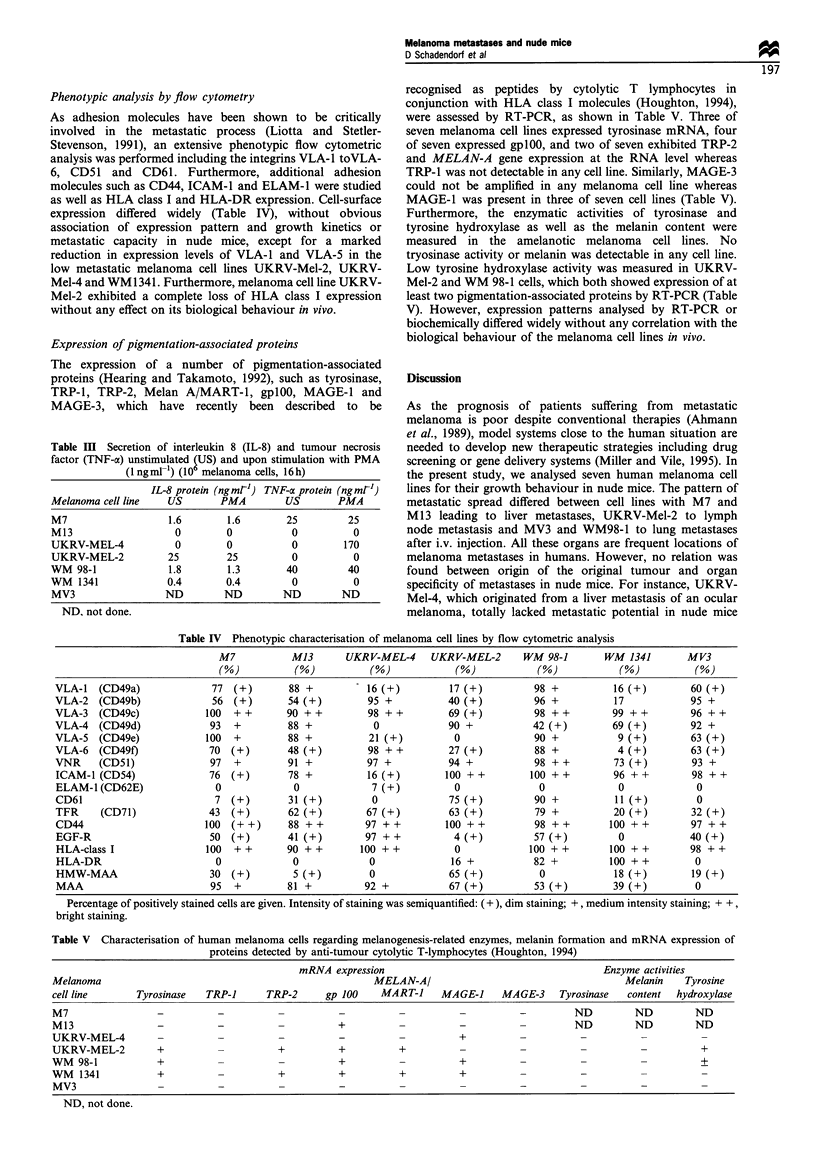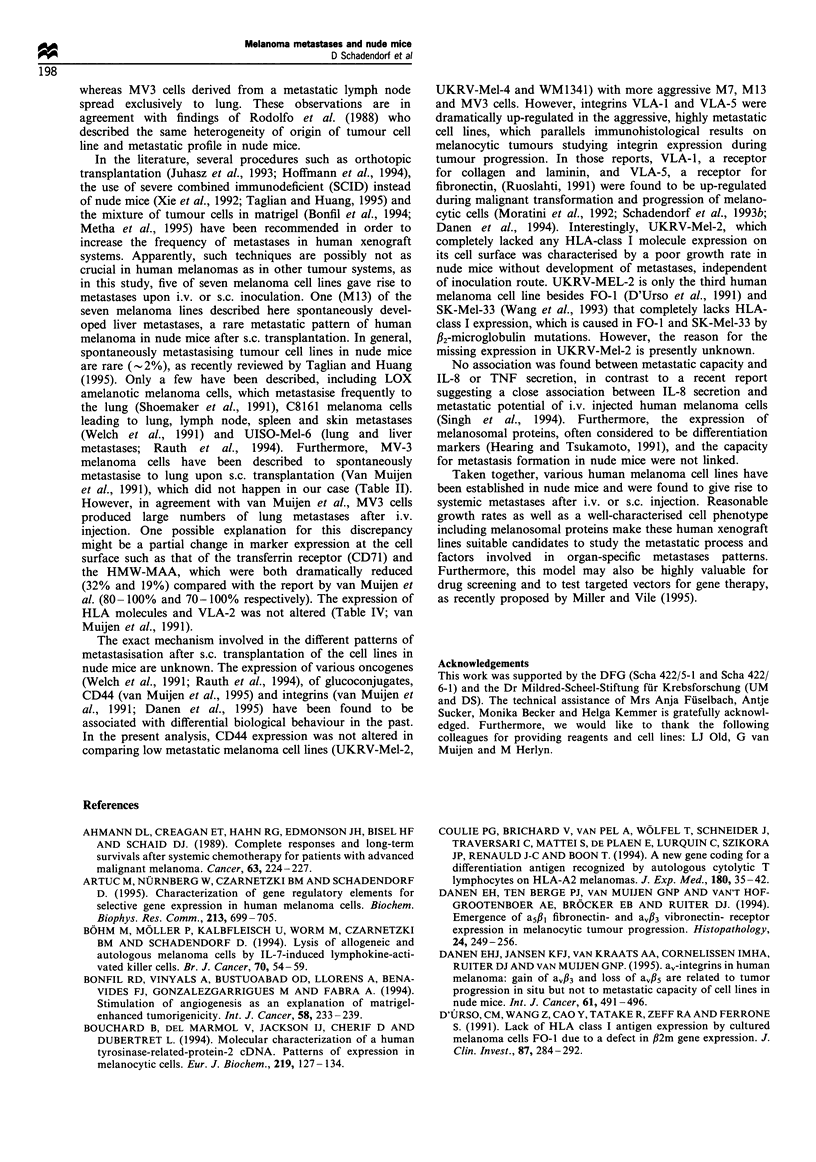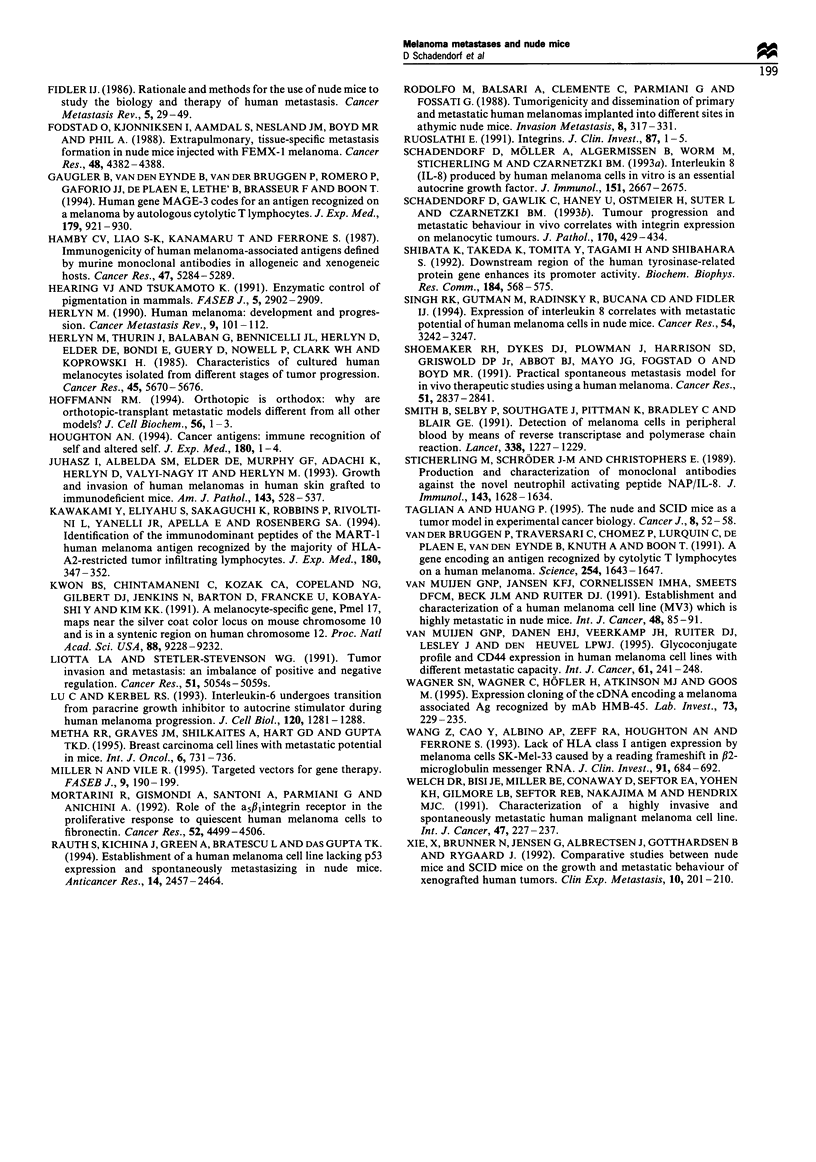# Metastatic potential of human melanoma cells in nude mice--characterisation of phenotype, cytokine secretion and tumour-associated antigens.

**DOI:** 10.1038/bjc.1996.337

**Published:** 1996-07

**Authors:** D. Schadendorf, I. Fichtner, A. Makki, S. Alijagic, M. Küpper, U. Mrowietz, B. M. Henz

**Affiliations:** Virchow Clinic, Department of Dermatology, Humboldt University of Berlin, Germany.

## Abstract

Incidence and mortality of human malignant melanoma has risen rapidly over recent decades. Although the notorious resistance to treatment is characteristic for metastatic malignant melanoma, only a few experimental models have been established to study the metastatic cascade or to test new alternative treatment modalities. Thus, new human models are wanted. Here, we describe the metastatic behaviour of seven human melanoma cell lines derived from two primary cutaneous melanomas (WM 98-1, WM 1341) and five metastases established from liver (UKRV-Mel-4), skin (M7, M13), pleural effusion (UKRV-Mel-2) and lymph node (MV3). All cell lines were analysed for their capacity to grow in nude mice after s.c. and i.v. administration. M13 cells developed liver metastases spontaneously after s.c. injection, and subsequent passages of M13 and M7 melanoma cells caused liver metastases after i.v. injection, whereas MV3 and WM98-1 gave rise to lung metastases, using the same inoculation route. In contrast, WM 1341, UKRV-Mel-2 and UKRV-Mel-4 grew only very slowly in nude mice after s.c. injection and did not cause any metastases after i.v. or s.c. administration. The pattern of metastases or growth kinetics did not correlate with the interleukin 8 or tumour necrosis factor secretion of cell lines. Adhesion molecules and growth factor receptor expression on the cell lines differed widely, as determined by flow cytometry, with the low metastatic cell lines (UKRV-Mel-2, UKRV-Mel-4 and WM 1341) demonstrating a marked reduction in VLA-1 and VLA-5 expression compared with the metastatic lines (M7, M13, MV3 and WM 98-1). Expression of pigment-related proteins such as tyrosinase, TRP-1, TRP-2, Melan-A/MART-1, gp100, MAGE1 or MAGE-3 was not associated with growth and metastatic characteristics of the melanoma cell lines analysed. In conclusion, the established human melanoma cell lines exhibited diverse growth behaviour in nude mice in congruence with some early established prognostic markers such as VLA-1 and VLA-5. The xenografts provide good models for further study of metastatic processes as well as for evaluation of alternative treatment modalities including new pharmaceutical drugs and gene therapeutic targeting using tissue-specific gene regulatory elements for gene targeting.


British Journal of Cancer (1996) 74, 194–199
© 1996 Stockton Press All rights reserved 0007–0920/96 $12.00

### Metastatic potential of human melanoma cells in nude mice – characterisation of phenotype, cytokine secretion and tumour-associated antigens

D Schadendorf[1], I Fichtner[2], A Makki[1], S Alijagic[1], M Küpper[3], U Mrowietz[3] and BM Henz[1]

[1]*Virchow Clinic, Department of Dermatology, Humboldt University of Berlin, 13344 Berlin;* [2]*Max Delbrück Center of Molecular Medicine, 13125 Berlin;* [3]*Department of Dermatology, University Clinic Kiel, 24105 Kiel, Germany.*

**Summary** Incidence and mortality of human malignant melanoma has risen rapidly over recent decades. Although the notorious resistance to treatment is characteristic for metastatic malignant melanoma, only a few experimental models have been established to study the metastatic cascade or to test new alternative treatment modalities. Thus, new human models are wanted. Here, we describe the metastatic behaviour of seven human melanoma cell lines derived from two primary cutaneous melanomas (WM 98-1, WM 1341) and five metastases established from liver (UKRV-Mel-4), skin (M7, M13), pleural effusion (UKRV-Mel-2) and lymph node (MV3). All cell lines were analysed for their capacity to grow in nude mice after s.c. and i.v. administration. M13 cells developed liver metastases spontaneously after s.c. injection, and subsequent passages of M13 and M7 melanoma cells caused liver metastases after i.v. injection, whereas MV3 and WM98-1 gave rise to lung metastases, using the same inoculation route. In contrast, WM 1341, UKRV-Mel-2 and UKRV-Mel-4 grew only very slowly in nude mice after s.c. injection and did not cause any metastases after i.v. or s.c. administration. The pattern of metastases or growth kinetics did not correlate with the interleukin 8 or tumour necrosis factor secretion of cell lines. Adhesion molecules and growth factor receptor expression on the cell lines differed widely, as determined by flow cytometry, with the low metastatic cell lines (UKRV-Mel-2, UKRV-Mel-4 and WM 1341) demonstrating a marked reduction in VLA-1 and VLA-5 expression compared with the metastatic lines (M7, M13, MV3 and WM 98-1). Expression of pigment-related proteins such as tyrosinase, TRP-1, TRP-2, Melan-A/MART-1, gp100, MAGE1 or MAGE-3 was not associated with growth and metastatic characteristics of the melanoma cell lines analysed. In conclusion, the established human melanoma cell lines exhibited diverse growth behaviour in nude mice in congruence with some early established prognostic markers such as VLA-1 and VLA-5. The xenografts provide good models for further study of metastatic processes as well as for evaluation of alternative treatment modalities including new pharmaceutical drugs and gene therapeutic targeting using tissue-specific gene regulatory elements for gene targeting.

**Keywords:** xenograft model; integrin expression; interleukin 8; spontaneous metastasis; liver metastases

Human melanoma in its advanced stage is characterised by frequent metastases to soft tissues such as skin, lung, lymph nodes and liver, with currently no hope for cure, as conventional therapeutic strategies are not effective (Ahmann *et al.*, 1989). Research on tumour progression, development of metastases and new therapeutic approaches such as new drugs or the use of tissue-specific promoter elements for gene targeting may open up alternative strategies for the treatment of metastatic melanoma. However, research is hampered by the lack of well-characterised models.

In order to mimic the human situation as closely as possible, transplants of human tumours in nude mice are increasingly used for the investigation of the metastatic process and the development of new therapeutic modalities (Fidler, 1986). Various human melanoma cell lines have been shown to grow in nude mice after s.c. inoculation or to cause organ metastases after i.v. administration (Fodstad *et al.*, 1988; van Muijen *et al.*, 1991; Shoemaker *et al.*, 1991; Welch *et al.*, 1991). However, only a few human melanoma cell lines give rise to spontaneous metastases in nude mice after s.c. implantation (Welch *et al.*, 1991; Shoemaker *et al.*, 1991; van Muijen *et al.*, 1991; Rauth *et al.*, 1994).

We report here the characteristics of seven human melanoma cell lines established from two primary cutaneous melanomas and five metastases derived from different tissues (liver, lymph node, skin and pleural effusion). Growth behaviour and metastatic capacity in nude mice were assessed after s.c. inoculation and after i.v. administration. Furthermore, melanoma cell lines were extensively characterised regarding several adhesion molecules, HLA molecules, pigment-associated proteins as well as the secretion of cytokines interleukin 8 (IL-8) and tumour necrosis factor (TNF-α), which have been shown to be correlated with metastatic capacity in nude mice (Singh *et al.*, 1994).

### Materials and methods

### *Cell culture*

Human melanoma cell lines WM 98-1 and WM1341 were derived from primary melanomas (Herlyn *et al.*, 1985; Herlyn, 1990; Lu and Kerbel, 1993) and were kindly provided by Dr M Herlyn (Wistar Institute, USA). MV3 human melanoma cells were established from a metastatic melanoma lymph node (van Muijen *et al.*, 1991) and were kindly provided by Dr van Muijen (Nijmegen, The Netherlands). M7 and M13 are newly established human melanoma cell lines derived from cutaneous metastases. UKRV-Mel-2 (derived from metastatic pleural effusion) and UKRV-Mel-4 (established from a liver metastasis of an ocular melanoma) were recently described by our group (Artuc *et al.*, 1995). Details regarding tumour and patient characteristics are given in Table I. All cell lines were maintained and cultured in RPMI-1640 medium supplemented with 10% fetal calf serum (FCS) and antibiotics as described previously (Schadendorf *et al.*, 1993*a*).

Correspondence: D Schadendorf, Virchow Clinic, HU Berlin, Department of Dermatology, Augustenburger Platz 1, 13344 Berlin, Germany
Received 4 December 1995; revised 14 February 1996; accepted 20 February 1996

*Polymerase chain reaction (PCR)*

PCR was carried out with reversely transcribed cDNA generated from all melanoma cell lines as described previously (Schadendorf *et al.*, 1993*a*). Primer sequences were devised according to the published sequence for human tyrosinase (Smith *et al.*, 1991), for MAGE-1 (Van der Bruggen *et al.*, 1991), for MAGE-3 (Gaugler *et al.*, 1994), for TRP-1 (Shibata *et al.*, 1992), for TRP-2 (Bouchard *et al.*, 1994), for gp100 (Kwon *et al.*, 1991; Wagner *et al.*, 1995) and for MELAN-A/MART-1 (Coulie *et al.*, 1994; Kawakami *et al.*, 1994). The following primers were used: Tyr-1: TTG GCA GAT TGT CTG TAG CC and Tyr-2: AGG CAT TGT GCA TGC TGC TT, which generate a 284 bp DNA amplificate specific for tyrosinase; Tyr-3: GTC TTT ATG CAA TGG AAC GC and Tyr-4: GCT ATC CCA GTA AGT GGA CT generate a second 207 bp DNA amplificate specific for tyrosinase; MAGE1-5: TTG CCG AAG ATC TCA GGA A and MAGE1-3: CTT GCC TCC TCA CAG AG generate a 470 bp DNA amplificate specific for the *MAGE-1* gene; MAGE3-5: TGG AGG ACC AGA GGC CCC C and MAGE3-3: GGA CGA TTA TCA GGA GGC CTG C generate a 714 bp DNA amplificate specific for the *MAGE-3* gene; TRP-15: AAA GGA TTA GTA AAG GGT and TRP-13: CAT TCT GCT TGA AAT AAG generate a 670 bp DNA amplificate specific for the *TRP-1* gene; TRP-25: CTG GGT GCA GAG TCG GCC and TRP-23: ATT GGG CCC AAG CAG GCC generate a 300 bp DNA amplificate specific for the *TRP-2* gene; pMEl175: AGA TCC TGC AGG CTG TGC and pMel173: CAA TGG GAC AAG AGC AGA generate a 540 bp DNA amplificate specific for the *gp100/pMel17* gene; MART1-5: ACT GCT CAT CGG CTG TTG and MART1-3: TCA GCC ATG TCT CAG GTG generate a 265 bp DNA amplificate specific for the *MART-1/MELAN-A* gene.

*Flow cytometric analysis*

Cell staining was performed using mouse monoclonal antibodies, followed by FITC-conjugated, affinity purified, isotype-specific goat anti-mouse antibodies (Immunotech, Hamburg, Germany) as described previously (Böhm *et al.*, 1994). The samples were analysed on a Coulter Epics XL Flow Cytometry System (Miami, FL, USA). The following MAbs were used: B9.12.1 ($IgG_{2a}$, anti-HLA-A,B,C, Immunotech, Hamburg, Germany), B.9.12.2 ($IgG_{2b}$, anti-HLA-DR, Immunotech), 84H10 [$IgG_1$, anti-ICAM-1 (CD54)], 225.28 (IgG, anti-HMW-MAA, gift from Dr S Ferrone; Hamby *et al.*, 1987), 376.96 (IgG, anti-100 kd-MAA, gift from Dr S Ferrone; Hamby *et al.*, 1987), ENA-1 [IgG, anti-ELAM-1 (CD62E), Dianova, Hamburg, Germany], GRO-1 ($IgG_1$, anti-EGF-R, Dianova), YDJ 1.2.2. [$IgG_1$, anti-Tansferrin-R (CD71), Dianova], ROS 220 ($IgG_1$, anti-CD44s, Dianova), TS2/7 ($IgG_1$, anti-CDw49a, VLA-1, T Cell Science), Gi9 ($IgG_1$, anti-CDw49b, VLA-2, Dianova), P1B5 ($IgG_1$, anti-CDw49c, Gibco), HP2/1 ($IgG_1$, anti-CDw49d, Dianova), SAM-1 ($IgG_{2a}$, anti-CDw49e, Dianova), GoH3 ($IgG_{2a}$, anti-CDw49f, Dianova), AMF/7 ($IgG_1$, anti-CD51, Dianova), SZ21 [$IgG_1$,anti-CD61 ($\beta_3$), Dianova]. All samples were analysed using propidium iodide to exclude dead cells.

*Melanogenic activity assays*

Melanogenic activity of the seven different cell lines was measured by enzymatic assays using [$^{14}$C]tyrosine for melanin formation and [$^3$H]tyrosine for tyrosine hydroxylase activity as described previously (Artuc *et al.*, 1995). The tyrosine hydroxylase assay measures only the first enzymatic function of the tyrosinase. The melanin formation assay measures the complete reaction sequence and reflects the melanogenic activities of tyrosinase, TRP-1 and TRP-2 (Hearing and Tsukamoto, 1991). Briefly, cells were harvested with trypsin–EDTA, solubilised with 100 $\mu$l of 1% NP-40 in 0.05 M Tris-HCl, pH 7.2, for 60 min at 4°C, and centrifuged for 15 min at 15 000 r.p.m.

*Tyrosine hydroxylase assay*

Tyrosine hydroxylase activity was measured by monitoring conversion of L-[$^3$H]tyrosine to L-dopa and the production of $^3H_2O$ as described (Artuc *et al.*, 1995). Cell extracts (50 $\mu$g of protein) were incubated in 50 $\mu$l of a reaction mixture containing 0.25 mM L-tyrosine, 5 $\mu$Ci ml$^{-1}$ [$^3$H]tyrosine and 0.05 mM L-dopa in 0.1 M phosphate buffer (pH 6.8) for 2 h at 37°C. The reaction was terminated by addition of 0.4 ml of charcoal (Norit SG activated charcoal) [10% (w/v) in 1% trichloroacetic acid (TCA)] and counted for radioactivity in a $\beta$-counter. One unit of the tyrosine hydroxylase was defined as the amount of the enzyme that catalyses the hydroxylation of 1 $\mu$mol L-tyrosine min$^{-1}$.

*Melanin formation assay*

Aliquots (30 $\mu$l) of the extracts were used to measure melanin formation as described previously (Artuc *et al.*, 1995). Incubations were performed at 37°C in a final volume of 50 $\mu$l for 2 h as for tyrosine hydroxylase (same substrate and final concentrations of the same co-factors), with [$^{14}$C]tyrosine as the radioactive substrate at a concentration of 0.106 mM (sp. act.: 50 Ci mmol$^{-1}$). The reaction was stopped by the addition of 150 $\mu$l of a solution containing 6% TCA and 10 mM unlabelled tyrosine. The samples were passed through fibreglass filters, and the filters were washed three times with 3 ml of 0.1 M hydrochloric acid, dried and counted in a $\beta$-counter.

*Melanin content*

Melanin content of the cells was calculated from the absorption of the cellular extracts compared with a standard curve for synthetic melanin at 475 nm (Artuc *et al.*, 1995). To measure the extent of melanisation, cell extracts containing 200 $\mu$g of protein were heated at 90°C for 2 h in 1 ml of 0.1 N sodium hydroxide and centrifuged at 14 000 r.p.m. for 20 min. The optical density of the supernatant was then measured at 475 nm.

*Cytokine secretion*

For the determination of IL-8 and TNF-$\alpha$ secretion, melanoma cells were maintained as described above. Cells were grown to subconfluence, washed twice with phosphate-buffered saline (PBS) and incubated with RPMI-1640 without FCS in two parallel sets with and without phorbol–myristate–acetate (1 ng ml$^{-1}$) for 16 h at 37°C. Thereafter, aliquots of the cell-free supernatant were collected and analysed for their content of IL-8 and TNF-$\alpha$ protein by specific ELISA. Secreted IL-8 protein was measured using a sandwich ELISA employing two specific monoclonal antibodies as recently described (Sticherling *et al.*, 1989) and TNF-$\alpha$ protein was determined using a commercially available ELISA (R&D Systems, Bad Nauheim, Germany).

*Animals*

For the *in vivo* experiments, male NMR/I: nu/nu mice (Bomholtgaard, Ry, Denmark) weighing 20–25 g were used. Mice were held under sterile conditions at 24–26°C room temperature, 50% relative humidity and 12 h light–dark rhythm in laminar flow shelves receiving autoclaved food (Sniff, Soest, Germany) and bedding. The drinking water was filtered and acidified (pH 4.0).

In vivo *transplantation*

For transplantation, melanoma cells were harvested by trypsinisation (0.25% trypsin; Seromed, Berlin, Germany) from cell culture flasks and were washed twice with PBS. Subsequently, cells were injected either s.c. ($10^7$ cells per mouse) into the right flank or i.v. ($10^6$ cells per mouse) into the tail vein of 2–3 animals each).

Mice were visited daily, and growing tumours were measured twice weekly with a caliper-like instrument. Moribund mice or mice whose tumours reached a diameter of 15 mm were killed by cervical dislocation. Non-necrotic tissues of primary tumours or metastases were cut into pieces of 2–3 mm diameter and used for further s.c. passages (P1, P2). In some cases, tumours were mechanically disaggregated with a glass homogeniser, and the resulting cell suspension was injected i.v. ($10^6$ cells per mouse). Metastases were semiquantitatively evaluated in all organs as follows: +, single small metastatic nodules; + +, up to five metastatic nodules of 4–5 mm diameter; + + +, numerous nodules/ massive metastatic infiltration.

### Results

### *Growth and metastatic capacity in nude mice*

All seven human melanoma cell lines were administered s.c. ($10^7$ cells per mouse) and i.v. ($10^6$ cells per mouse). The time interval between s.c. injection and tumour development ranged between 8 and 86 days. The growth of melanoma metastases after i.v. administration took between 42 and 120 days (Table II). Three cell lines (M13, UKRV-Mel-4, WM 1341) did not generate any metastases after i.v. injection and UKRV-Mel-2 gave rise to a lymph node metastasis in one of three mice without success of further passing the tumour cells to further animals. In addition, those melanoma cell lines with no or low metastatic capacity after i.v. injection, such as M13, UKRV-Mel-4, WM 1341 and UKRV-Mel-2 grew into small cutaneous tumours only after s.c. administration. In contrast, WM 98.1 and MV3 reproducibly generated numerous lung metastases 120 and 55 days, respectively, after i.v. inoculation. M7 cells as well as M13 cells derived from a mouse liver metastasis ($M13_{P2liver}$) also led repeatedly to liver metastases after i.v. injection (Table II). Furthermore, M7, WM 98.1 and MV3 melanoma cells, which all demonstrated some metastatic capacity after i.v. administration, formed, in addition, massive cutaneous tumours upon s.c. application. However, in all cases, no spontaneous systemic metastases were detected. Only M13 melanoma cells originally derived from a cutaneous melanoma metastasis produced not only large cutaneous tumours, but also spontaneously induced liver metastases (Table II). Passage of these cells into further animals led to a subclone ($M13_{P2liver}$), which demonstrated a high capacity to generate repeatedly liver metastases independent of inoculation route (s.c. or i.v.) with a latency period of around 65 days.

### *Secretion of IL-8 and TNF-α*

As cytokines, particularly IL-8, have been thought to influence the proliferation of human melanoma cells *in vitro* (Schadendorf *et al.*, 1993*a*) and to alter the metastatic and growth behaviour in nude mice (Singh *et al.*, 1994), we analysed the expression of IL-8 and TNF-α on RNA level by reverse transcriptase-polymerase chain reaction (RT-PCR) and on protein level by ELISA. IL-8 protein levels ranged between 0 and 25 ng $ml^{-1}$ per 100 000 cells 16 $h^{-1}$ independent of stimulation with phorbol 12-myristate 13-acetate (PMA) (Table III). Similarly, TNF-α was detectable in two of six (unstimulated) and in three of six (PMA-treated) cell lines, with protein levels between 0 and 170 ng $ml^{-1}$ per 100 000 cells 16 $h^{-1}$. IL-8 and TNF-α secretion levels *in vitro* were not associated with origin of tumour and with the capacity to grow or metastasise in nude mice.

**Table I** Origin of melanoma cell lines established on nude mice

| *Melanoma cell line* | *Source of metastasis* | *Gender* | *Tumour type* | *Tumour thickness* | *Localisation of PT* | *Reference* |
|---|---|---|---|---|---|---|
| M7 | Cutaneous | Male | NM | 5.00 mm | Neck | Unpublished |
| M13 | Cutaneous | Female | NM | 7.0 mm | Arm | Unpublished |
| UKRV-MEL-4 | Liver | Female | Ocular | – | Eye | Artuc *et al.* (1995) |
| UKRV-MEL-2 | Pleural effusion | Female | SSM | 1.8 mm | Arm | Artuc *et al.* (1995) |
| WM 98-1 | Primary melanoma | ? | SSM | ? | ? | Herlyn *et al.* (1985) |
| WM 1341 | Primary melanoma | ? | SSM | ? | ? | Herlyn (1990) |
| MV3 | Lymph node | Male | NM | 60 mm | Chin | van Muijen *et al.* (1991) |

PT, primary tumour.

**Table II** Establishment of human melanoma cell lines on nude mice. Determination of tumour take, tumour growth after s.c. inoculation ($10^7$ cells per mouse) and development of metastases after s.c. or i.v. injection of melanoma cells ($10^6$ cells)

| *Melanoma cell line* | *Inoculation site* | *Number of mice (n)* | *Latency in days Primary tumour* | *Metastases* | *Description of tumour take and metastases* |
|---|---|---|---|---|---|
| M7 | s.c. | 3 | 34 | | 3/3 massive cutaneous tumours; no metastases |
| | i.v. | 3 | | 100 | 1/3 liver metastases + +; ascites |
| M13 | s.c. | 3 | 34 | 42 | 1/3 liver metastases + +; ascites |
| | i.v. | 3 | | 90+ | Mice alive, no metastases |
| M13 P2 liver | s.c. | 2 | 30 | 71 | 2/2 liver metastases + + |
| | i.v. | 2 | | 64 | 1/2 liver metastases + |
| UKRV-MEL-4 | s.c. | 3 | 22 | | 2/2 small cutaneous tumours, mice alive, no metastases |
| | i.v. | 3 | | 100+ | Mice alive, no metastases |
| UKRV-MEL-2 | s.c. | 3 | 8 | | 3/3 cutaneous tumors; mice alive; no metastases |
| | i.v. | 3 | | 85 | 1/3 lymph node metastases + |
| WM 98-1 | s.c. | 3 | 39 | | 3/3 cutaneous tumors; no metastases |
| | i.v. | 3 | | 120 | 3/3 lung metastases + + + + |
| WM 1341 | s.c. | 3 | 86 | | 2/2 small cutaneous tumours, no metastases |
| | i.v. | 3 | | 150+ | Mice alive, no metastases |
| MV 3 | s.c. | 3 | 22 | | Massive cutaneous tumors–no metastases |
| | i.v. | 3 | | 55–85 | 3/3 lung metastases + + + + |
| MV P2 lung | i.v. | 3 | | 54 | 3/3 lung metastases + + + + |

*Phenotypic analysis by flow cytometry*

As adhesion molecules have been shown to be critically involved in the metastatic process (Liotta and Stetler-Stevenson, 1991), an extensive phenotypic flow cytometric analysis was performed including the integrins VLA-1 toVLA-6, CD51 and CD61. Furthermore, additional adhesion molecules such as CD44, ICAM-1 and ELAM-1 were studied as well as HLA class I and HLA-DR expression. Cell-surface expression differed widely (Table IV), without obvious association of expression pattern and growth kinetics or metastatic capacity in nude mice, except for a marked reduction in expression levels of VLA-1 and VLA-5 in the low metastatic melanoma cell lines UKRV-Mel-2, UKRV-Mel-4 and WM1341. Furthermore, melanoma cell line UKRV-Mel-2 exhibited a complete loss of HLA class I expression without any effect on its biological behaviour *in vivo*.

*Expression of pigmentation-associated proteins*

The expression of a number of pigmentation-associated proteins (Hearing and Takamoto, 1992), such as tyrosinase, TRP-1, TRP-2, Melan A/MART-1, gp100, MAGE-1 and MAGE-3, which have recently been described to be recognised as peptides by cytolytic T lymphocytes in conjunction with HLA class I molecules (Houghton, 1994), were assessed by RT-PCR, as shown in Table V. Three of seven melanoma cell lines expressed tyrosinase mRNA, four of seven expressed gp100, and two of seven exhibited TRP-2 and *MELAN-A* gene expression at the RNA level whereas TRP-1 was not detectable in any cell line. Similarly, MAGE-3 could not be amplified in any melanoma cell line whereas MAGE-1 was present in three of seven cell lines (Table V). Furthermore, the enzymatic activities of tyrosinase and tyrosine hydroxylase as well as the melanin content were measured in the amelanotic melanoma cell lines. No tryosinase activity or melanin was detectable in any cell line. Low tyrosine hydroxylase activity was measured in UKRV-Mel-2 and WM 98-1 cells, which both showed expression of at least two pigmentation-associated proteins by RT-PCR (Table V). However, expression patterns analysed by RT-PCR or biochemically differed widely without any correlation with the biological behaviour of the melanoma cell lines *in vivo*.

**Table III** Secretion of interleukin 8 (IL-8) and tumour necrosis factor (TNF-α) unstimulated (US) and upon stimulation with PMA (1 ng ml$^{-1}$) (10$^6$ melanoma cells, 16 h)

| | *IL-8 protein (ng ml$^{-1}$)* | | *TNF-α protein (ng ml$^{-1}$)* | |
|---|---|---|---|---|
| *Melanoma cell line* | *US* | *PMA* | *US* | *PMA* |
| M7 | 1.6 | 1.6 | 25 | 25 |
| M13 | 0 | 0 | 0 | 0 |
| UKRV-MEL-4 | 0 | 0 | 0 | 170 |
| UKRV-MEL-2 | 25 | 25 | 0 | 0 |
| WM 98-1 | 1.8 | 1.3 | 40 | 40 |
| WM 1341 | 0.4 | 0.4 | 0 | 0 |
| MV3 | ND | ND | ND | ND |

ND, not done.

### Discussion

As the prognosis of patients suffering from metastatic melanoma is poor despite conventional therapies (Ahmann *et al.*, 1989), model systems close to the human situation are needed to develop new therapeutic strategies including drug screening or gene delivery systems (Miller and Vile, 1995). In the present study, we analysed seven human melanoma cell lines for their growth behaviour in nude mice. The pattern of metastatic spread differed between cell lines with M7 and M13 leading to liver metastases, UKRV-Mel-2 to lymph node metastasis and MV3 and WM98-1 to lung metastases after i.v. injection. All these organs are frequent locations of melanoma metastases in humans. However, no relation was found between origin of the original tumour and organ specificity of metastases in nude mice. For instance, UKRV-Mel-4, which originated from a liver metastasis of an ocular melanoma, totally lacked metastatic potential in nude mice

**Table IV** Phenotypic characterisation of melanoma cell lines by flow cytometric analysis

| | *M7 (%)* | *M13 (%)* | *UKRV-MEL-4 (%)* | *UKRV-MEL-2 (%)* | *WM 98-1 (%)* | *WM 1341 (%)* | *MV3 (%)* |
|---|---|---|---|---|---|---|---|
| VLA-1 (CD49a) | 77 (+) | 88 + | 16 (+) | 17 (+) | 98 + | 16 (+) | 60 (+) |
| VLA-2 (CD49b) | 56 (+) | 54 (+) | 95 + | 40 (+) | 96 + | 17 | 95 + |
| VLA-3 (CD49c) | 100 + + | 90 + + | 98 + + | 69 (+) | 98 + + | 99 + + | 96 + + |
| VLA-4 (CD49d) | 93 + | 88 + | 0 | 90 + | 42 (+) | 69 (+) | 92 + |
| VLA-5 (CD49e) | 100 + | 88 + | 21 (+) | 0 | 90 + | 9 (+) | 63 (+) |
| VLA-6 (CD49f) | 70 (+) | 48 (+) | 98 + + | 27 (+) | 88 + | 4 (+) | 63 (+) |
| VNR (CD51) | 97 + | 91 + | 97 + | 94 + | 98 + + | 73 (+) | 93 + |
| ICAM-1 (CD54) | 76 (+) | 78 + | 16 (+) | 100 + + | 100 + + | 96 + + | 98 + + |
| ELAM-1 (CD62E) | 0 | 0 | 7 (+) | 0 | 0 | 0 | 0 |
| CD61 | 7 (+) | 31 (+) | 0 | 75 (+) | 90 + | 11 (+) | 0 |
| TFR (CD71) | 43 (+) | 62 (+) | 67 (+) | 63 (+) | 79 + | 20 (+) | 32 (+) |
| CD44 | 100 (+ +) | 88 + + | 97 + + | 100 + + | 98 + + | 100 + + | 97 + + |
| EGF-R | 50 (+) | 41 (+) | 97 + + | 4 (+) | 57 (+) | 0 | 40 (+) |
| HLA-class I | 100 + + | 90 + + | 100 + + | 0 | 100 + + | 100 + + | 98 + + |
| HLA-DR | 0 | 0 | 0 | 16 + | 82 + | 100 + + | 0 |
| HMW-MAA | 30 (+) | 5 (+) | 0 | 65 (+) | 0 | 18 (+) | 19 (+) |
| MAA | 95 + | 81 + | 92 + | 67 (+) | 53 (+) | 39 (+) | 0 |

Percentage of positively stained cells are given. Intensity of staining was semiquantified: (+), dim staining; +, medium intensity staining; + +, bright staining.

**Table V** Characterisation of human melanoma cells regarding melanogenesis-related enzymes, melanin formation and mRNA expression of proteins detected by anti-tumour cytolytic T-lymphocytes (Houghton, 1994)

| | *mRNA expression* | | | | | | | *Enzyme activities* | | |
|---|---|---|---|---|---|---|---|---|---|---|
| *Melanoma cell line* | *Tyrosinase* | *TRP-1* | *TRP-2* | *gp 100* | *MELAN-A/ MART-1* | *MAGE-1* | *MAGE-3* | *Tyrosinase* | *Melanin content* | *Tyrosine hydroxylase* |
| M7 | – | – | – | – | – | – | – | ND | ND | ND |
| M13 | – | – | – | + | – | – | – | ND | ND | ND |
| UKRV-MEL-4 | – | – | – | – | – | + | – | – | – | – |
| UKRV-MEL-2 | + | – | + | + | + | – | – | – | – | + |
| WM 98-1 | + | – | – | + | – | + | – | – | – | ± |
| WM 1341 | + | – | + | + | + | + | – | – | – | – |
| MV3 | – | – | – | – | – | – | – | – | – | – |

ND, not done.

whereas MV3 cells derived from a metastatic lymph node spread exclusively to lung. These observations are in agreement with findings of Rodolfo *et al.* (1988) who described the same heterogeneity of origin of tumour cell line and metastatic profile in nude mice.

In the literature, several procedures such as orthotopic transplantation (Juhasz *et al.*, 1993; Hoffmann *et al.*, 1994), the use of severe combined immunodeficient (SCID) instead of nude mice (Xie *et al.*, 1992; Taglian and Huang, 1995) and the mixture of tumour cells in matrigel (Bonfil *et al.*, 1994; Metha *et al.*, 1995) have been recommended in order to increase the frequency of metastases in human xenograft systems. Apparently, such techniques are possibly not as crucial in human melanomas as in other tumour systems, as in this study, five of seven melanoma cell lines gave rise to metastases upon i.v. or s.c. inoculation. One (M13) of the seven melanoma lines described here spontaneously developed liver metastases, a rare metastatic pattern of human melanoma in nude mice after s.c. transplantation. In general, spontaneously metastasising tumour cell lines in nude mice are rare (~2%), as recently reviewed by Taglian and Huang (1995). Only a few have been described, including LOX amelanotic melanoma cells, which metastasise frequently to the lung (Shoemaker *et al.*, 1991), C8161 melanoma cells leading to lung, lymph node, spleen and skin metastases (Welch *et al.*, 1991) and UISO-Mel-6 (lung and liver metastases; Rauth *et al.*, 1994). Furthermore, MV-3 melanoma cells have been described to spontaneously metastasise to lung upon s.c. transplantation (Van Muijen *et al.*, 1991), which did not happen in our case (Table II). However, in agreement with van Muijen *et al.*, MV3 cells produced large numbers of lung metastases after i.v. injection. One possible explanation for this discrepancy might be a partial change in marker expression at the cell surface such as that of the transferrin receptor (CD71) and the HMW-MAA, which were both dramatically reduced (32% and 19%) compared with the report by van Muijen *et al.* (80–100% and 70–100% respectively). The expression of HLA molecules and VLA-2 was not altered (Table IV; van Muijen *et al.*, 1991).

The exact mechanism involved in the different patterns of metastasisation after s.c. transplantation of the cell lines in nude mice are unknown. The expression of various oncogenes (Welch *et al.*, 1991; Rauth *et al.*, 1994), of glucoconjugates, CD44 (van Muijen *et al.*, 1995) and integrins (van Muijen *et al.*, 1991; Danen *et al.*, 1995) have been found to be associated with differential biological behaviour in the past. In the present analysis, CD44 expression was not altered in comparing low metastatic melanoma cell lines (UKRV-Mel-2, UKRV-Mel-4 and WM1341) with more aggressive M7, M13 and MV3 cells. However, integrins VLA-1 and VLA-5 were dramatically up-regulated in the aggressive, highly metastatic cell lines, which parallels immunohistological results on melanocytic tumours studying integrin expression during tumour progression. In those reports, VLA-1, a receptor for collagen and laminin, and VLA-5, a receptor for fibronectin, (Ruoslahti, 1991) were found to be up-regulated during malignant transformation and progression of melanocytic cells (Moratini *et al.*, 1992; Schadendorf *et al.*, 1993*b*; Danen *et al.*, 1994). Interestingly, UKRV-Mel-2, which completely lacked any HLA-class I molecule expression on its cell surface was characterised by a poor growth rate in nude mice without development of metastases, independent of inoculation route. UKRV-MEL-2 is only the third human melanoma cell line besides FO-1 (D'Urso *et al.*, 1991) and SK-Mel-33 (Wang *et al.*, 1993) that completely lacks HLA-class I expression, which is caused in FO-1 and SK-Mel-33 by $\beta_2$-microglobulin mutations. However, the reason for the missing expression in UKRV-Mel-2 is presently unknown.

No association was found between metastatic capacity and IL-8 or TNF secretion, in contrast to a recent report suggesting a close association between IL-8 secretion and metastatic potential of i.v. injected human melanoma cells (Singh *et al.*, 1994). Furthermore, the expression of melanosomal proteins, often considered to be differentiation markers (Hearing and Tsukamoto, 1991), and the capacity for metastasis formation in nude mice were not linked.

Taken together, various human melanoma cell lines have been established in nude mice and were found to give rise to systemic metastases after i.v. or s.c. injection. Reasonable growth rates as well as a well-characterised cell phenotype including melanosomal proteins make these human xenograft lines suitable candidates to study the metastatic process and factors involved in organ-specific metastases patterns. Furthermore, this model may also be highly valuable for drug screening and to test targeted vectors for gene therapy, as recently proposed by Miller and Vile (1995).

**Acknowledgements**

This work was supported by the DFG (Scha 422/5-1 and Scha 422/6-1) and the Dr Mildred-Scheel-Stiftung für Krebsforschung (UM and DS). The technical assistance of Mrs Anja Füselbach, Antje Sucker, Monika Becker and Helga Kemmer is gratefully acknowledged. Furthermore, we would like to thank the following colleagues for providing reagents and cell lines: LJ Old, G van Muijen and M Herlyn.

### References

AHMANN DL, CREAGAN ET, HAHN RG, EDMONSON JH, BISEL HF AND SCHAID DJ. (1989). Complete responses and long-term survivals after systemic chemotherapy for patients with advanced malignant melanoma. *Cancer*, **63**, 224–227.

ARTUC M, NÜRNBERG W, CZARNETZKI BM AND SCHADENDORF D. (1995). Characterization of gene regulatory elements for selective gene expression in human melanoma cells. *Biochem. Biophys. Res. Comm.*, **213**, 699–705.

BÖHM M, MÖLLER P, KALBFLEISCH U, WORM M, CZARNETZKI BM AND SCHADENDORF D. (1994). Lysis of allogeneic and autologous melanoma cells by IL-7-induced lymphokine-activated killer cells. *Br. J. Cancer*, **70**, 54–59.

BONFIL RD, VINYALS A, BUSTUOABAD OD, LLORENS A, BENAVIDES FJ, GONZALEZGARRIGUES M AND FABRA A. (1994). Stimulation of angiogenesis as an explanation of matrigel-enhanced tumorigenicity. *Int. J. Cancer*, **58**, 233–239.

BOUCHARD B, DEL MARMOL V, JACKSON IJ, CHERIF D AND DUBERTRET L. (1994). Molecular characterization of a human tyrosinase-related-protein-2 cDNA. Patterns of expression in melanocytic cells. *Eur. J. Biochem.*, **219**, 127–134.

COULIE PG, BRICHARD V, VAN PEL A, WÖLFEL T, SCHNEIDER J, TRAVERSARI C, MATTEI S, DE PLAEN E, LURQUIN C, SZIKORA JP, RENAULD J-C AND BOON T. (1994). A new gene coding for a differentiation antigen recognized by autologous cytolytic T lymphocytes on HLA-A2 melanomas. *J. Exp. Med.*, **180**, 35–42.

DANEN EH, TEN BERGE PJ, VAN MUIJEN GNP AND VAN'T HOF-GROOTENBOER AE, BRÖCKER EB AND RUITER DJ. (1994). Emergence of $a_5\beta_1$ fibronectin- and $a_v\beta_3$ vibronectin- receptor expression in melanocytic tumour progression. *Histopathology*, **24**, 249–256.

DANEN EHJ, JANSEN KFJ, VAN KRAATS AA, CORNELISSEN IMHA, RUITER DJ AND VAN MUIJEN GNP. (1995). $a_v$-integrins in human melanoma: gain of $a_v\beta_3$ and loss of $a_v\beta_5$ are related to tumor progression in situ but not to metastatic capacity of cell lines in nude mice. *Int. J. Cancer*, **61**, 491–496.

D'ÚRSO, CM, WANG Z, CAO Y, TATAKE R, ZEFF RA AND FERRONE S. (1991). Lack of HLA class I antigen expression by cultured melanoma cells FO-1 due to a defect in $\beta$2m gene expression. *J. Clin. Invest.*, **87**, 284–292.

FIDLER IJ. (1986). Rationale and methods for the use of nude mice to study the biology and therapy of human metastasis. *Cancer Metastasis Rev.*, **5**, 29–49.
FODSTAD O, KJONNIKSEN I, AAMDAL S, NESLAND JM, BOYD MR AND PHIL A. (1988). Extrapulmonary, tissue-specific metastasis formation in nude mice injected with FEMX-1 melanoma. *Cancer Res.*, **48**, 4382–4388.
GAUGLER B, VAN DEN EYNDE B, VAN DER BRUGGEN P, ROMERO P, GAFORIO JJ, DE PLAEN E, LETHE' B, BRASSEUR F AND BOON T. (1994). Human gene MAGE-3 codes for an antigen recognized on a melanoma by autologous cytolytic T lymphocytes. *J. Exp. Med.*, **179**, 921–930.
HAMBY CV, LIAO S-K, KANAMARU T AND FERRONE S. (1987). Immunogenicity of human melanoma-associated antigens defined by murine monoclonal antibodies in allogeneic and xenogeneic hosts. *Cancer Res.*, **47**, 5284–5289.
HEARING VJ AND TSUKAMOTO K. (1991). Enzymatic control of pigmentation in mammals. *FASEB J.*, **5**, 2902–2909.
HERLYN M. (1990). Human melanoma: development and progression. *Cancer Metastasis Rev.*, **9**, 101–112.
HERLYN M, THURIN J, BALABAN G, BENNICELLI JL, HERLYN D, ELDER DE, BONDI E, GUERY D, NOWELL P, CLARK WH AND KOPROWSKI H. (1985). Characteristics of cultured human melanocytes isolated from different stages of tumor progression. *Cancer Res.*, **45**, 5670–5676.
HOFFMANN RM. (1994). Orthotopic is orthodox: why are orthotopic-transplant metastatic models different from all other models? *J. Cell Biochem.*, **56**, 1–3.
HOUGHTON AN. (1994). Cancer antigens: immune recognition of self and altered self. *J. Exp. Med.*, **180**, 1–4.
JUHASZ I, ALBELDA SM, ELDER DE, MURPHY GF, ADACHI K, HERLYN D, VALYI-NAGY IT AND HERLYN M. (1993). Growth and invasion of human melanomas in human skin grafted to immunodeficient mice. *Am. J. Pathol.*, **143**, 528–537.
KAWAKAMI Y, ELIYAHU S, SAKAGUCHI K, ROBBINS P, RIVOLTINI L, YANELLI JR, APELLA E AND ROSENBERG SA. (1994). Identification of the immunodominant peptides of the MART-1 human melanoma antigen recognized by the majority of HLA-A2-restricted tumor infiltrating lymphocytes. *J. Exp. Med.*, **180**, 347–352.
KWON BS, CHINTAMANENI C, KOZAK CA, COPELAND NG, GILBERT DJ, JENKINS N, BARTON D, FRANCKE U, KOBAYASHI Y AND KIM KK. (1991). A melanocyte-specific gene, Pmel 17, maps near the silver coat color locus on mouse chromosome 10 and is in a syntenic region on human chromosome 12. *Proc. Natl Acad. Sci. USA*, **88**, 9228–9232.
LIOTTA LA AND STETLER-STEVENSON WG. (1991). Tumor invasion and metastasis: an imbalance of positive and negative regulation. *Cancer Res.*, **51**, 5054s–5059s.
LU C AND KERBEL RS. (1993). Interleukin-6 undergoes transition from paracrine growth inhibitor to autocrine stimulator during human melanoma progression. *J. Cell Biol.*, **120**, 1281–1288.
METHA RR, GRAVES JM, SHILKAITES A, HART GD AND GUPTA TKD. (1995). Breast carcinoma cell lines with metastatic potential in mice. *Int. J. Oncol.*, **6**, 731–736.
MILLER N AND VILE R. (1995). Targeted vectors for gene therapy. *FASEB J.*, **9**, 190–199.
MORTARINI R, GISMONDI A, SANTONI A, PARMIANI G AND ANICHINI A. (1992). Role of the $a_5\beta_1$integrin receptor in the proliferative response to quiescent human melanoma cells to fibronectin. *Cancer Res.*, **52**, 4499–4506.
RAUTH S, KICHINA J, GREEN A, BRATESCU L AND DAS GUPTA TK. (1994). Establishment of a human melanoma cell line lacking p53 expression and spontaneously metastasizing in nude mice. *Anticancer Res.*, **14**, 2457–2464.
RODOLFO M, BALSARI A, CLEMENTE C, PARMIANI G AND FOSSATI G. (1988). Tumorigenicity and dissemination of primary and metastatic human melanomas implanted into different sites in athymic nude mice. *Invasion Metastasis*, **8**, 317–331.
RUOSLATHI E. (1991). Integrins. *J. Clin. Invest.*, **87**, 1–5.
SCHADENDORF D, MÖLLER A, ALGERMISSEN B, WORM M, STICHERLING M AND CZARNETZKI BM. (1993*a*). Interleukin 8 (IL-8) produced by human melanoma cells in vitro is an essential autocrine growth factor. *J. Immunol.*, **151**, 2667–2675.
SCHADENDORF D, GAWLIK C, HANEY U, OSTMEIER H, SUTER L AND CZARNETZKI BM. (1993*b*). Tumour progression and metastatic behaviour in vivo correlates with integrin expression on melanocytic tumours. *J. Pathol.*, **170**, 429–434.
SHIBATA K, TAKEDA K, TOMITA Y, TAGAMI H AND SHIBAHARA S. (1992). Downstream region of the human tyrosinase-related protein gene enhances its promoter activity. *Biochem. Biophys. Res. Comm.*, **184**, 568–575.
SINGH RK, GUTMAN M, RADINSKY R, BUCANA CD AND FIDLER IJ. (1994). Expression of interleukin 8 correlates with metastatic potential of human melanoma cells in nude mice. *Cancer Res.*, **54**, 3242–3247.
SHOEMAKER RH, DYKES DJ, PLOWMAN J, HARRISON SD, GRISWOLD DP Jr, ABBOT BJ, MAYO JG, FOGSTAD O AND BOYD MR. (1991). Practical spontaneous metastasis model for in vivo therapeutic studies using a human melanoma. *Cancer Res.*, **51**, 2837–2841.
SMITH B, SELBY P, SOUTHGATE J, PITTMAN K, BRADLEY C AND BLAIR GE. (1991). Detection of melanoma cells in peripheral blood by means of reverse transcriptase and polymerase chain reaction. *Lancet*, **338**, 1227–1229.
STICHERLING M, SCHRÖDER J-M AND CHRISTOPHERS E. (1989). Production and characterization of monoclonal antibodies against the novel neutrophil activating peptide NAP/IL-8. *J. Immunol.*, **143**, 1628–1634.
TAGLIAN A AND HUANG P. (1995). The nude and SCID mice as a tumor model in experimental cancer biology. *Cancer J.*, **8**, 52–58.
VAN DER BRUGGEN P, TRAVERSARI C, CHOMEZ P, LURQUIN C, DE PLAEN E, VAN DEN EYNDE B, KNUTH A AND BOON T. (1991). A gene encoding an antigen recognized by cytolytic T lymphocytes on a human melanoma. *Science*, **254**, 1643–1647.
VAN MUIJEN GNP, JANSEN KFJ, CORNELISSEN IMHA, SMEETS DFCM, BECK JLM AND RUITER DJ. (1991). Establishment and characterization of a human melanoma cell line (MV3) which is highly metastatic in nude mice. *Int. J. Cancer*, **48**, 85–91.
VAN MUIJEN GNP, DANEN EHJ, VEERKAMP JH, RUITER DJ, LESLEY J AND DEN HEUVEL LPWJ. (1995). Glycoconjugate profile and CD44 expression in human melanoma cell lines with different metastatic capacity. *Int. J. Cancer*, **61**, 241–248.
WAGNER SN, WAGNER C, HÖFLER H, ATKINSON MJ AND GOOS M. (1995). Expression cloning of the cDNA encoding a melanoma associated Ag recognized by mAb HMB-45. *Lab. Invest.*, **73**, 229–235.
WANG Z, CAO Y, ALBINO AP, ZEFF RA, HOUGHTON AN AND FERRONE S. (1993). Lack of HLA class I antigen expression by melanoma cells SK-Mel-33 caused by a reading frameshift in $\beta$2-microglobulin messenger RNA. *J. Clin. Invest.*, **91**, 684–692.
WELCH DR, BISI JE, MILLER BE, CONAWAY D, SEFTOR EA, YOHEN KH, GILMORE LB, SEFTOR REB, NAKAJIMA M AND HENDRIX MJC. (1991). Characterization of a highly invasive and spontaneously metastatic human malignant melanoma cell line. *Int. J. Cancer*, **47**, 227–237.
XIE, X, BRUNNER N, JENSEN G, ALBRECTSEN J, GOTTHARDSEN B AND RYGAARD J. (1992). Comparative studies between nude mice and SCID mice on the growth and metastatic behaviour of xenografted human tumors. *Clin Exp. Metastasis*, **10**, 201–210.